# DNA-COMPACT: DNA COMpression Based on a Pattern-Aware Contextual Modeling Technique

**DOI:** 10.1371/journal.pone.0080377

**Published:** 2013-11-25

**Authors:** Pinghao Li, Shuang Wang, Jihoon Kim, Hongkai Xiong, Lucila Ohno-Machado, Xiaoqian Jiang

**Affiliations:** 1 Division of Biomedical Informatics, University of California San Diego, La Jolla, California, United States of America; 2 Department of Electronic Engineering, Shanghai Jiao Tong University, Shanghai, China; The Centre for Research and Technology, Hellas, Greece

## Abstract

Genome data are becoming increasingly important for modern medicine. As the rate of increase in DNA sequencing outstrips the rate of increase in disk storage capacity, the storage and data transferring of large genome data are becoming important concerns for biomedical researchers. We propose a two-pass lossless genome compression algorithm, which highlights the synthesis of complementary contextual models, to improve the compression performance. The proposed framework could handle genome compression with and without reference sequences, and demonstrated performance advantages over best existing algorithms. The method for reference-free compression led to bit rates of 1.720 and 1.838 bits per base for bacteria and yeast, which were approximately 3.7% and 2.6% better than the state-of-the-art algorithms. Regarding performance with reference, we tested on the first Korean personal genome sequence data set, and our proposed method demonstrated a 189-fold compression rate, reducing the raw file size from 2986.8 MB to 15.8 MB at a comparable decompression cost with existing algorithms. DNAcompact is freely available at https://sourceforge.net/projects/dnacompact/for research purpose.

## Introduction

Massively parallel sequencing (MPS) is leading revolutionary advances in understanding of health and disease in humans. It produces far more sequencing reads at a significantly lower cost than conventional techniques such as Sanger-based capillary sequencing, which contributed to the Human Genome Project that released the first human DNA sequence in 2001 [1]. Each human has two complementary copies of 3.2 Gigabases. The 1000 Genomes Project [2] has produced more than fifty TeraBytes (TB) of data with 1,092 individuals from fourteen populations, toward the goal of sequencing 2,500 individuals in total [3]. The data size of the Sequence Read Archive, an international public archival resource of sequence reads, is expecting to exceed 1000 Terabases by the end of 2013 (http://www.ncbi.nlm.nih.gov/Traces/sra/sra.cgi?view=announcement/). The sequencing output is doubling every nine months, surpassing the performance improvements of computation and storage [4]. Data compression methods for reducing the storage space and saving the data transfer bandwidth are becoming crucial for the efficient management of large genome data.

Two previous studies [5], [6] have classified genome compression problem into three categories based on the data type: (1) unaligned short reads in FASTQ format (e.g. Quip [7], G-SQZ [8], SCALCE [9] and DSRC [10]); (2) aligned short reads in BAM format (e.g. CRAM [11], SlimGene [5], SAMZIP [12], and NGC [6]); and (3) assembly in FASTA format (e.g. XM [13], RLZ [14], GRS [15], compression with the Burrows-Wheeler transform [16] and GReEn [17]). In this paper, we focus on the third category and study the compression issue in two scenarios, i.e., without and with a reference sequence.

De novo sequencing, where the reference sequence is unavailable, is getting a lot of attention from the biomedical community. One such example is in metagenomics [18], where combined metagenome is enormous even though individual genome size might be small. In the realm of reference-free genome data compression, two categories of approaches, *dictionary-based algorithms* and *statistics-based algorithms*, are used to tackle this problem. Most dictionary-based methods search for repeating subsequences (including forward and reverse complements) and encode them by referring a previous subsequence with maximum matching length. The most representative works include the first dedicated DNA compression algorithm Biocompress [19] and CTW+LZ [20]. Alternatively, researchers also use low-order Markov models to encode regions [21]–[24] when the substitutional methods perform unfavorably. The statistical coding algorithms been evolving: the rudimentary second-order arithmetic encoding [24], the normalized maximum likelihood (NML) algorithm [25], the expert-model (XM) algorithm [13] and the state-of-the-art FCM-C [26] algorithm. NML aims at finding the best regressor block, i.e., approximate repetition or first-order dependencies that have not been considered in the substitutional approaches. XM relies on a mixture of experts to provide symbol-by-symbol probability estimates that are used to drive an arithmetic encoder (AE). FCM-C uses two competing finite-context models to capture different aspects of statistical information along the sequence.

Due to the fact that the nucleotide diversity within the same species is relatively small (e.g., the difference in humans is around 0.1% [27], i.e., one difference per 1,000 base pairs), most recent improvements in genome compression models are reference-based, such as RLZ [14], GRS [15] and GReEn [17]. RLZ indexes the reference sequence and applies the relative Lempel-Ziv algorithm. GRS applies the Huffman algorithm after checking the differential rate between the target and reference sequences. GReEn is based on arithmetic coding that relies on the copy model, where the pointers to the reference sequence position (highly likely conserved ones) are encoded to generate the probability distribution of the symbols. All these existing reference-based DNA compression algorithms apply identical schemes to the mapped regions that can find the repeats in the reference sequence and unmapped regions, which lead to a source of redundancy in the compressed file.

**Figure 1:**
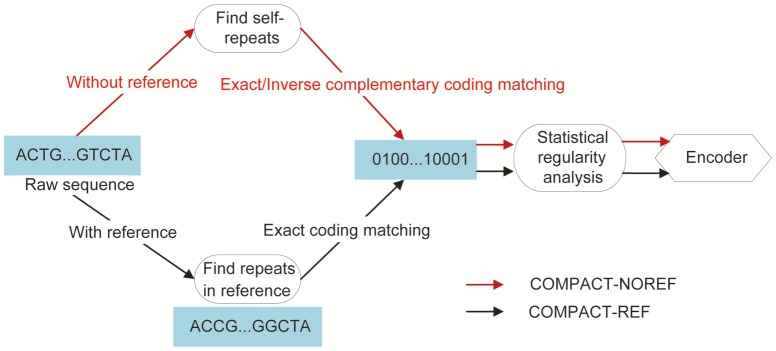
Overview for two versions of DNA COMpression based on Pattern-Aware Contextual modeling Technique (COMPACT). In the first pass, the COMPACT-NOREF scheme aims to search self-similarity and complimentary palindromes within raw sequence, while the COMPACT-REF scheme explores the sparse representation of the target sequences in terms of the reference sequence. Both schemes share the second pass to discriminate statistical regularities.

Essentially, all compression methods have to make compromise on the trade-off between compression ratio and complexity. We setup our target applications to a practical scenario, where disk space is the limiting factor but compression time is relatively more tolerable. We propose a novel two-pass lossless DNA compression framework to take advantage of dictionary-based and statistics-based algorithms to deal with the genome compression for scenarios with and without reference sequences. A high level overview is illustrated in Figure 1.

In the first pass of compression without references (COMPACT-NOREF), we search exact repeats within the raw sequence as well as complementary palindromes and represent them by a compact quadruplet. This is slightly different in the first pass of compression with references (COMPACT-REF), in which we study variations between the target sequences and reference sequences from the same species. Both COMPACT-NOREF and COMPACT-REF share the same second pass. Our main contributions are two-fold. First, we introduce non-sequential contextual models to capture non-sequential characteristics within DNA sequences, which are not considered in the existing DNA compression methods. Second, contrary to methods XM [13] and FCM-Mx [28] that combine contextual models by Bayesian averaging and its modifications, our approach for synthesizing contextual models is less likely to produce biased results (As pointed out by Minka [29], Bayesian averaging tends to favor only one model among many with MAP (i.e., Maximum A Posteriori) estimation, while logistic regression assigns ‘’weights’’ as the expression of the ‘’appropriateness’’ of all candidate hypotheses to make full use of the information that is available).

## Materials and Methods

### Experimental Materials

We used a public dataset of DNA sequences in Table 1, which has been used in many other DNA compression publications (ftp://ftp.infotech.monash.edu.au/ftp/ftp/software/DNAcompress-XM/XMCompress/dataSet/). The material (www.mfn.unipmn.it/manzini/dnacorpus) showed in Table 2 is the same as the one used by Manzini et al. in dnaX [30]. This corpus contains sequences from four organisms: yeast (*Saccharomyces cerevisiae*, chromosomes 1, 4, 14 and the mitochondrial DNA), mouse (*Mus musculus*, chromosomes 7, 11, 19, X and Y), arabidopsis (*Arabidopsis thaliana*, chromosomes 1, 3 and 4) and human (*Homo sapiens*, chromosomes 2, 13, 22, X and Y). In our experiments, we also used the bacteria DNA sequences (Table 3) collected from the National Center for Biotechnology Information (NCBI) directory (ftp://ftp.ncbi.nih.gov/genomes/Bacteria/). In addition, complete DNA sequences of eleven species of various sizes were also used [31]–[41].

**Table 1:** Comparison of DNA compression performance on a standard dataset.

Seq.	DNA3	XM500	FCM	COMPACT-seq	COM-NOREF			Default
	(2004)	(2007)	(2009)		dnaX	LZ	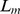	
CHMPXX	1.6782	1.6598	**1.6276**	1.6491	1.6490	1.6470	50	1.6532
CHNTXX	1.6223	1.6088	1.6270	1.6046	1.6027	**1.6008**	18	1.6063
HEHCMVCG	1.8463	1.8426	1.8472	1.8386	**1.8072**	1.8185	18	1.8076
HUMDYSTROP	1.9533	**1.9031**	1.9258	1.9190	1.9202	**1.9086**	18	1.9295
HUMHBB	1.8807	**1.7543**	1.8650	1.8309	1.8291	1.8254	50	1.8359
MPOMTCG	1.9312	**1.8826**	1.9213	1.9021	1.9029	1.8944	30	1.9067
MTPACG	1.8735	1.8487	1.8641	1.8448	1.8439	**1.8393**	30	1.8491
VACCG	1.7645	1.7659	1.7697	1.7577	1.7553	**1.7534**	18	1.7591
Average.	1.8182	**1.7832**	1.8060	1.7933	1.7881	**1.7859**	-	1.7934

Note: Values in each column (except for 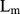) refer to bit per base (bpb). 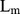 refers to the respective minimum match length in the first pass and ‘Default’ column represents the compression results of COM-NONREF with default parameter 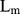 (i.e., 25). The ‘’XM500’’ refers to the XM algorithm using at most 500 experts. ‘’COMPACT-seq’’ indicates one mode of our proposed method that uses only traditional sequential models followed by logistic regression model. The value of the best performance model is in bold font.

**Table 2:** Compression of DNA compression performance on four organisms.

Sequence	Size	DNA3	XM500	FCM	COMPACT			Default
	(bytes)	(2004)	(2007)	(2009)	-seq	-NOREF	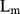	
y-1	230,203	1.871	**1.8103**	1.860	1.8478	1.8432	15	1.8552
y-4	1,531,929	1.881	1.8687	1.879	1.8719	**1.8682**	20	1.8710
y-14	784,328	1.926	1.9141	1.923	1.9154	**1.9114**	20	1.9136
y-mit	85,779	1.523	1.4714	1.484	**1.4305**	**1.4309**	40	1.4515
Average	--	1.882	**1.8642**	1.877	1.8684	1.8648	--	1.8686
m-7	5,114,647	1.835	**1.7259**	1.811	1.7933	1.7842	80	1.8029
m-11	49,909,125	1.790	**1.6759**	1.758	1.7584	1.7528	40	1.7637
m-19	703,729	1.888	**1.8126**	1.870	1.8368	1.8355	50	1.8509
m-x	17,430,763	1.703	**1.5235**	1.656	1.6653	1.6463	100	1.6780
m-y	711,108	1.707	**1.4904**	1.670	1.6600	1.6570	100	1.6696
Average	--	1.772	**1.6429**	1.738	1.7386	1.7297	--	1.7461
at-1	29,830,437	1.844	**1.7495**	1.831	1.8270	1.8239	60	1.8199
at-3	23,465,336	1.843	**1.7297**	1.826	1.8263	1.8220	50	1.8183
at-4	17,550,033	1.851	**1.7527**	1.838	1.8386	1.8313	100	1.8263
Average	--	1.845	**1.7437**	1.831	1.8296	1.8251	--	1.8209
h-2	236,268,154	1.790	1.632*	1.755	1.7554	1.7359	100	1.7661
h-13	95,206,001	1.818	1.664*	1.723	1.7849	1.7677	100	1.8134
h-22	33,821,688	1.767	**1.594**3	1.696	1.6824	1.6705	60	1.7423
h-x	144,793,946	1.732	1.493*	1.686	1.6923	1.6652	150	1.7042
h-y	22,668,225	1.411	**1.1251**	1.397	1.3746	1.3555	100	1.3732
Average	--	1.762	**1.5760**	1.711	1.7227	**1.7020**	--	1.7395

Note: Values in each column (except for Size and 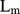) refer to bits per base (bpb). ‘*’ indicates that a model consumes much more memory (around or larger than 2.0GB) than other methods. The proposed algorithm COMPACT-NOREF provides a best result among all other algorithms (except ‘XM’).

**Table 3:** Individual compression results on the five sequences of bacteria.

Sequence	XM500 (2007)		FCM-Mx (2011)		Semi -COMPACT		COMPACT -seq		COMPACT NOREF	
	Time	bpb	Time	bpb	Time	bpb	Time	bpb	Time	bpb
NC_013929	288	1.786	71	1.754	340	1.7269	140	1.7588	310	**1.7153**
NC_014318	296	1.789	73	1.739	350	1.7083	150	1.7496	313	**1.7025**
NC_013595	312	1.796	73	1.759	350	1.7268	150	1.7651	319	**1.7189**
NC_013131	323	1.817	75	1.779	352	1.7397	160	1.7857	319	**1.7366**
NC_010162	371	1.755	92	1.743	438	1.7296	170	1.7443	386	**1.7230**
Average	1.7870		1.7543		1.7265		1.7600		**1.7204**	

Note: The minimum match length 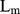 in the first pass is set to 25 in this experiment. The unit of time is second and ‘bpb’ refers to bits per base. Results of ‘’FCM-Mx’’ are obtained from ([28]) as we could not obtain the software to test in our own computer.

For compression with a reference sequence, we used the same data as those listed in [17], [15] to perform a fair comparison with GRS [15] and GReEn [17]. These data include two versions of the individual Korean genome sequences, KOREF_20090131 and KOREF_20090224 [42]. Note that the genome of a Han Chinese is referred to as YH [43]. The human genome reference assembly hg18 was released from the UCSC Genome Browser.

### Algorithm Description

**Figure 2:**
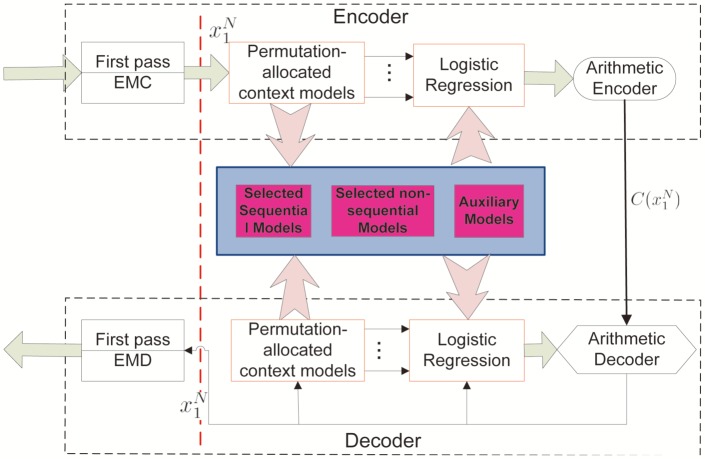
Our proposed two-pass DNA compression framework. The first pass is designed to maximally reduce repetitions. The second pass handles statistical regularities in the non-repetition zones by synthesizing a mixture of contextual models. ‘’EMC’’ refers to ‘’Exact Matching Coding’’ and ‘’EMD’’ refers to ‘’Exact Matching Decoding’’.

The proposed two-pass framework for DNA compression is depicted in Figure 2. In this section, we start with the description of the proposed first pass, which focuses on substitution-based exact matching coding (EMC). For the second pass, we introduce our pattern-aware contextual modeling technique.

### The first pass

The goal of this pass is to remove redundancy, such as repetitions, reverse complements (a.k.a., complemented palindromes), etc. Because COMPACT was designed to handle genome data compression with and without references, we will discuss the first pass algorithms for each scenario, separately.

**Figure 3:**
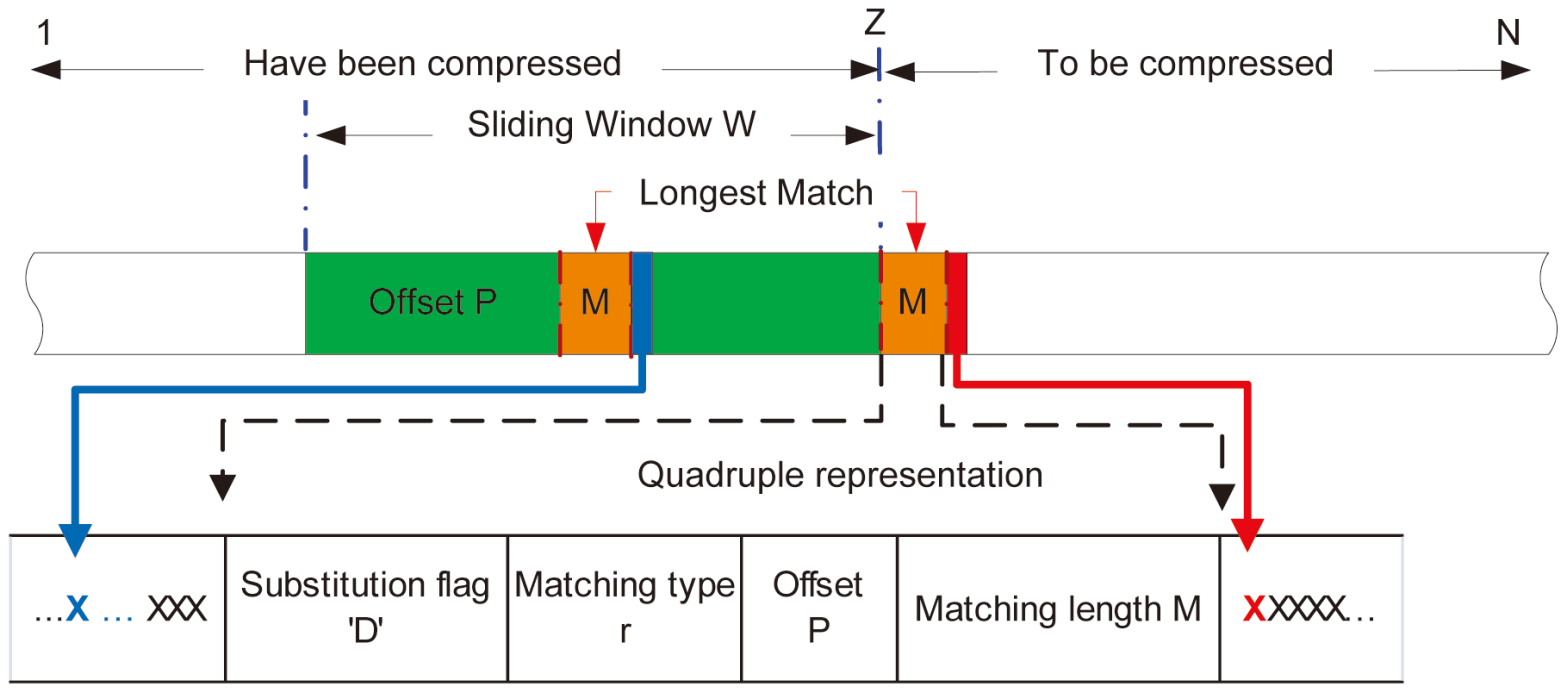
The diagram of the proposed first-pass algorithm, where the longest match with length 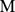 is represented by a quadruplet 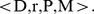 given a sliding window. The uncertainty of the symbol 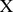 in red (i.e., the next symbol to be compressed) is reduced with the information provided by the symbol 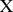 in blue (i.e., the neighboring symbol after the longest match in the sliding window).

**Without reference.** Figure 3 illustrates how the first-pass algorithm works in situations without a reference. Like the typical compression algorithm Lempel-Ziv (i.e., LZ) ([44]), suppose the initial portion 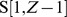 of the input sequence has been compressed, and 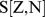 is the remaining sequence to be compressed, where 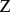 indicates the 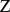-th symbol and
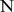
is the total length of the input sequence. We denote the portion
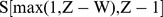
as the search window for the remaining sequence 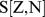, where 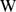
is the predefined sliding window size. The algorithm compares substrings within
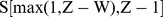
and 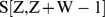
to find the longest match 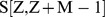
(such that L≤M≤W), where 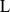 is a threshold parameter (we will discuss the ‘’optimal parameter’’ in the *Results* section).

As illustrated in Figure 3, each repeat is represented by a quadruplet 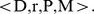
Substitution flag ‘
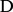
’ is encoded in the second pass with other DNA bases, so it is not discussed here. Matching type 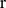 requires only one bit, which has no need of compression. We concentrated on the encoding of the offset position 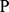 and the matching length 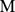. Our algorithm calculated the offset position 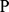 as the distance (i.e., number of symbols) between the first symbol of matching and the beginning of the search window (see Figure 3).

We used a log-skewed encoding mechanism [30] to store the offset position 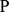
because: (1) the average code length of such method is no more than 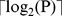 bits, and (2) the method assigns fewer bits for smaller 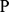 when possible. Regarding the longest matching length 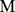, we encoded the value of 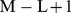 instead of 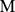 (because 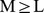) for better coding efficiency, using a Gamma coding mechanism [45] that writes the value's binary representation preceded by 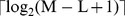 zeros. We replaced each subsequence that has a satisfying match with the corresponding encoded quadruplet, and sent unmatched bases to the second pass COMPACT coder for further processing.

**Figure 4:**
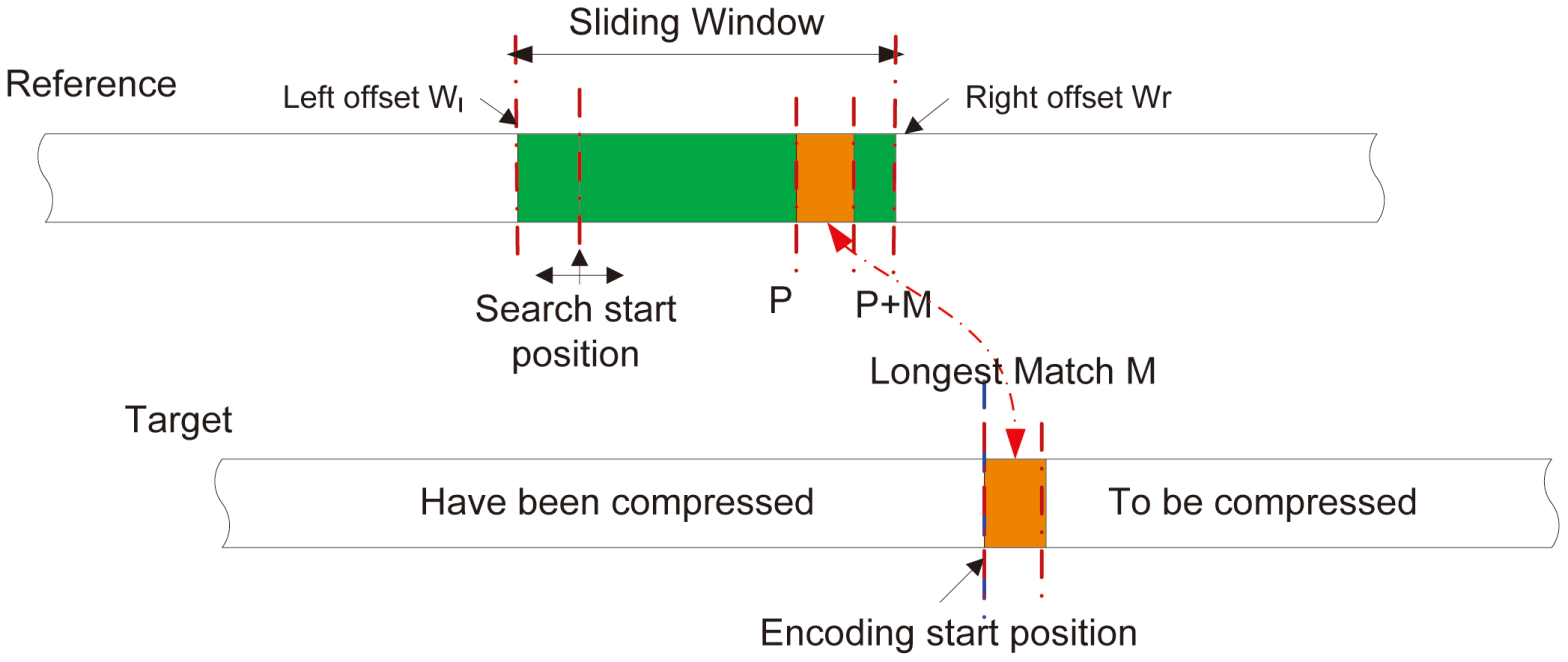
The diagram for the proposed algorithm rLZ. We define the range of the sliding window as 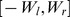.

**With reference.** We used an adaptive mechanism (denoted by rLZ) to compress genomes when reference sequences are available. Similar to the aforementioned first pass in the reference-free compression, rLZ treats subsequences of the reference sequence as the sliding window, and conducts bi-directionally searches from the starting position for the longest and nearest exact repeats of the current DNA fragment in the target sequence (see Figure 4). The bi-directional search ensures the tracking of substitution, insertion, and deletion at a close range between the target sequence and the reference sequence.

Different from the scenario for compression without reference, the match length 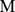
is usually much longer and the offset value 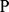 is much smaller. Hence, we encoded the match length 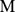 by log-skewed coding while encoding the offset value 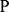 using Gamma coding. Likewise, the remaining bases after substitution or insertion will be sent to the second pass.

### The second pass

**Figure 5:**
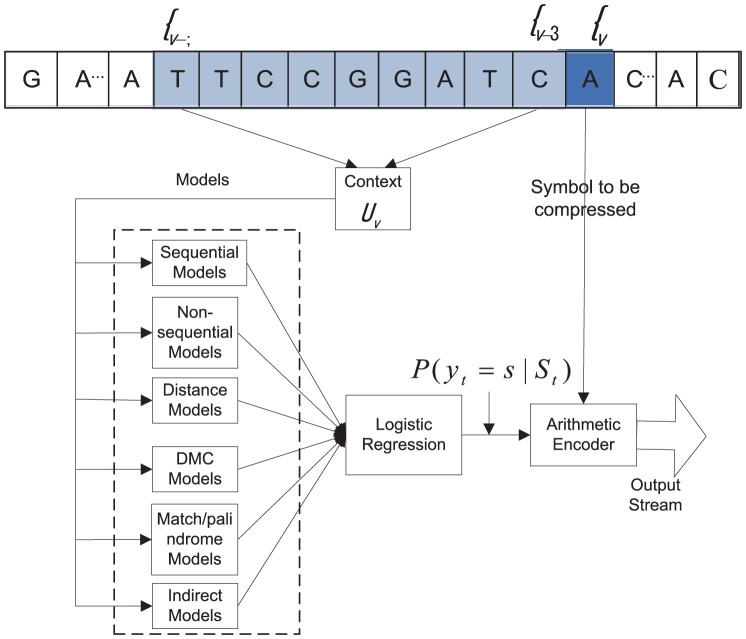
The framework of the second pass. Multiple context models (e.g., sequential models, non-sequential models, etc.) are combined by logistic regression to feed an arithmetic encoder for compression. ‘’DMC’’ is the abbreviation of ‘’Dynamic Markov Compression’’.

After the first pass, the remaining uncoded sequence will be further compressed through our second pass, in which each contextual model provides a probability given certain prior knowledge of the symbol/bit to be encoded, as shown in Figure 5. Then, the logistic regression model will synthesize these contextual models’ predictions. The eventual output will be sent to an arithmetic encoder (i.e., a form of entropy encoding that encodes the entire message into a single message).

**Figure 6:**
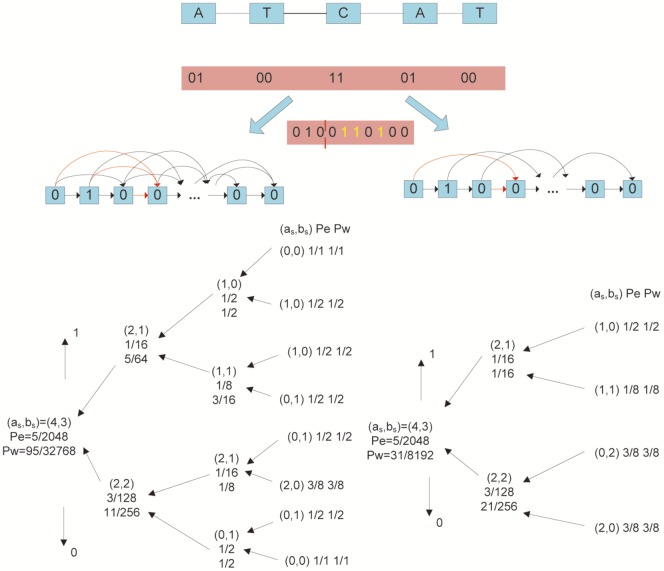
Weighted context tree for DNA bit sequence 0100110100 (ATCAT, T-00, A-01, G-10, C-11). (a, Left) The context tree is created according to a third order sequential context set. The code length generated for the sequence is 
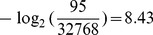
 bits; (b, Right) The context tree is made with the corresponding model excluding the second symbol (i.e., a non-sequential model). The code length generated for the sequence is 
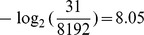
 bits.

The example shown in Figure 6 offers an intuitive way to show the advantage of non-sequential contextual models [46]. Suppose that the alphabet of DNA sequence complies with the following mappings, i.e., T-00, A-01, G-10, C-11. Then, for example, a given DNA sequence ATCAT in Figure 6 can be represented by its corresponding binary form as 0100110100. The left side of Figure 6 shows a 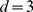 order sequential contextual model for the given binary DNA sequence, where each bit 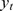 at location 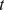 depends on the previous three bits, i.e., 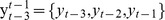 (the red link lines are an example). In contrast, a 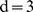 order non-sequential contextual model can be found in the right side of Figure 6, where each bit 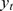 only depends on the bits 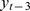 and 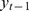. Furthermore, we define the dependency of the DNA bit as the context denoted by 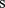. For example, both 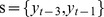 and 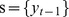 are the contexts of 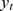 in our non-sequential model. We also define 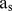 and 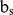 as the number of bit 0's and bit 1's with the context 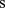 in a given DNA sequence, for which we will not assign any context for the first 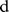 bits in the sequence. For instance, the number of bit 0’s and bit 1’s with context 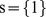
(the bits with yellow color) in the left side of Figure 6 is 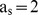 and 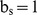. Finally, we denote by 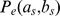
the estimated probability under corresponding context, and use 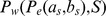 to represent the weighted probabilities over a set of contexts 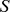 (i.e.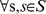). Readers can check the CTW algorithm in ([47]-[48]) for more details about procedures for calculating 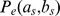 and 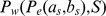. In the example illustrated in Figure 6, the probability 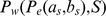 obtained through a non-sequential model is much higher than that of a sequential model. As a higher 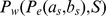 will result in a better compression performance (in an arithmetic coder), we expect the non-sequential model have greater prediction power in this case.

Given a DNA sequence 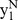, one of the non-sequential models for such sequence can be defined as 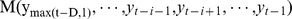
Where 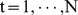 and each bit 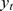 depends on its previous 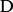 bits excluding the 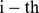
one (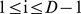). Similarly, the sequential model of the same sequence can be expressed as 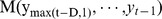.

We denote by 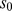 and 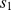 the contexts that satisfy the aforementioned sequential model with the 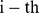
bit equaling 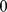 and 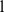, respectively. The context 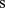 of our non-sequential model is almost the same as 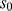
and 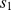 except for that omitted 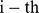
bit. We further denote by 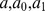 and 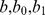 the number of bit 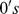 and 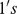 under the given contexts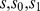, respectively. Then, the ratios between the corresponding counts can be expressed as 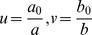 and 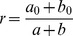. The following proposition, which was proved by Dai *et. al.* in [46], gives the sufficient condition by which a non-sequential model can outperform a sequential one, under the following assumptions: (1) the difference between 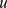 and 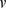 is small and 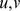 are not close to zero or one; (2) the ratio factor 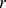 is close to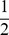; (3) the sequence length 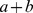 is large enough.

**Proposition 1.** Under the KT-estimator [47], the non-sequential context model will result in a better coding efficiency than a sequential context model when the distribution (i.e., the counts of bit 1’s and 0’s) of the corresponding context satisfies the following inequality. 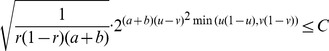
(1)where 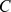 is a constant near the value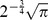.

### Pattern-Aware Contextual Modeling:

Although non-sequential models have advantages under certain conditions, in reality, sequential and non-sequential context models are complementary, which should be taken into consideration for completeness. However, to make computation feasible, we have to use only a small sample of the available context models. First, we define the foremost label of the selected symbols ahead of the base including the bit to be compressed as the context order ^n^, e.g., the context order is 6 when the context is selected as
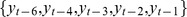
for 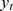. According to previous context-based DNA algorithms [26], the sequential context models with competing order (e.g., a low order and a high order) have significant effect on the final compression performance. Hence, when the maximum context order in the proposed method is set to 16, our models specifically consist of eleven general sequential context models with orders equal 1, 2, 4, 6, 8, 10, 11, 12, 13, 14, 16, a total of eleven non^—^sequential sparse models performed on the last four bytes (before the base including the bit to be compressed). If we use bit 1 to refer the picked bit and 0 for the excluded one, the eleven sparse models can be represented as ‘00F0F0F0’, ‘F0F0F0F0’, ‘00F8F8F8’, ‘F8F8F8F8’, ’00E0E0E0’, ‘E0E0E0E0’, ‘00F0F0FE’, ‘AAAAAAAA’, ‘F00F00FA’, ‘F000F0FD’ and ‘F0000F00’, which are series of hexadecimal digits (The reason why we choose these non-sequential contexts is explained in Appendix 5 of File S1 combined with Figure S3 in File S1).

For the 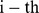
(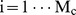) model in COMPACT, the prediction of the next outcome bit 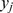 can be expressed as 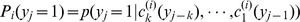, where 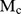 is the number of total context models, and 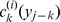, 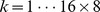
(i.e., the maximum context order multiplies the bit number of each byte) indicates the contextual dependencies (a.k.a. bit history) for 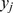 defined in the 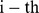
model. For example, 
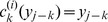
 if 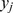 depends on 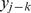 in the 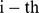
model, otherwise, 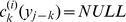. Then, 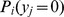 can be easily calculated as 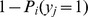. In our implementation, we apply three lookup-table based functions (i.e., Run Map, Stationary Map, and Nonstationary Map, referred to http://cs.fit.edu/mmahoney/compression/), to map the bit history to the corresponding probability.

In the rest of this paper, we denote the prediction result of bit 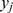 to be processed by the 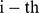 context model by 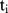 instead of 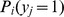. In the next section, we discuss how to find the most likely probability
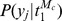
given the individual predictions 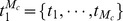 of all 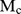 context models.

### Model synthesis based on logistic regression

According to the Maximum Entropy Principle (i.e. MAXENT [49]), the most likely probability for 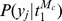 is the one with the highest entropy [50] as follows, 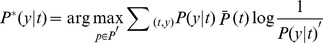
(2)where 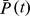 is the empirical probability of 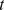 and 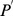 is subject to 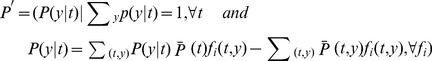
(3)


where 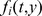 is a function that returns 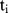 if 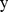 equals to the bit being predicted, or returns 0 otherwise. And 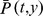 is the experienced probability of 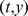. Eq.(2) with constrains of Eq.(3) can be transformed into the following equation through Lagrange multipliers. 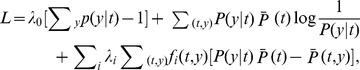
which gives 
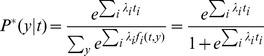
(4)By replacing the parameter 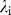 with 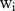, which can be viewed as the weight of the 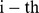 model, we obtain 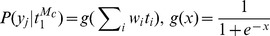
(5)


Given a binary bit 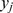, this is exactly a logistic regression model, which can be optimized efficiently using the Newton-Raphson algorithm. We add one diagram Figure S1 in File S1 to show how this model works in each step.

## Results

### Parameters discussion: model order 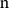 and minimum match length 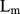


We implemented the encoder and decoder of COMPACT in C++, and ran experiments on a workstation with Intel(R) Xeon(R) 3.6 GHZ CPU and 96 GB of RAM. Denoting the parameters model order by 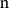 and minimum match length by 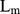 in the proposed algorithm, we explored the relationship between the compression performance (the average number of bits per base, bpb) and parameters. To study the issue comprehensively, we selected four sequences from different species with different sizes : HUMHPRTB, 56,737 symbols; HEHCMVCG, 229,354 symbols; y-4, 1,531,929 symbols; NC013929, 10,148,695 symbols.

**Figure 7:**
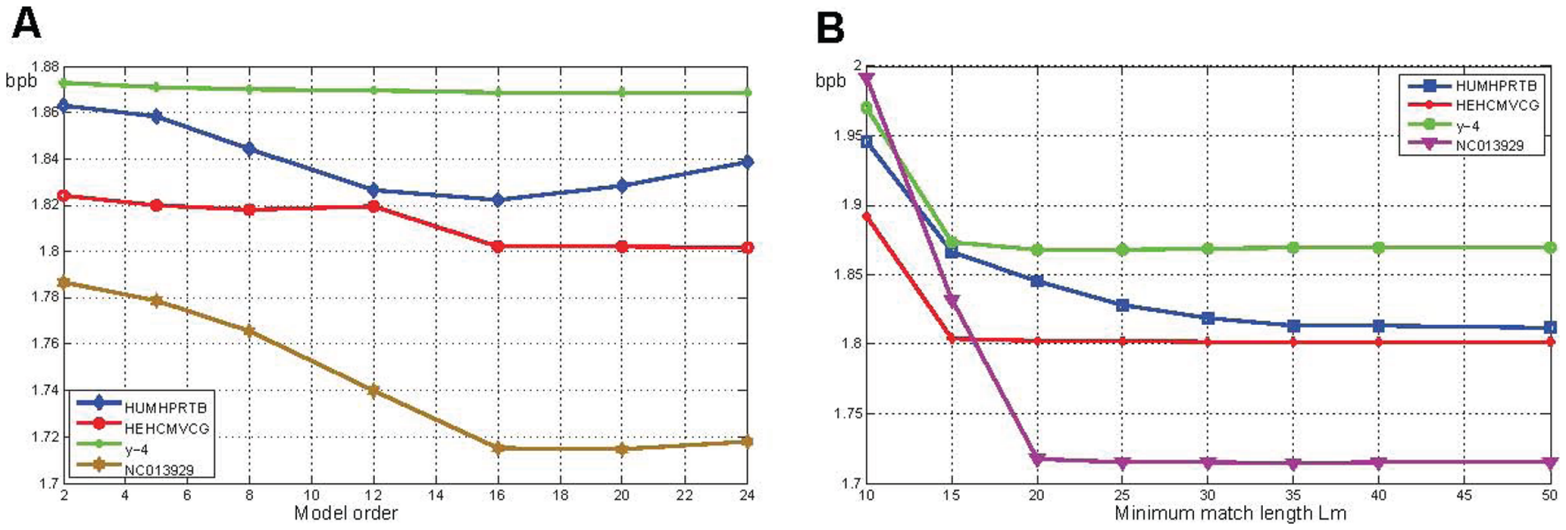
The relationship between the compression performance and COMPACT parameters. (a) model order n (using 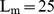) and (b) minimum match length 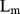 (using 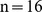).

As indicated in Figure 7, almost all sequences demonstrated a decrease in bits per base with the increase in model order. Correlation in the sequence was best predicted using context models of moderate orders. We chose 16 as the context model order. The figure also shows that the compression performance does not always improve with the growing of the minimum match length.

### Performance of compression without reference

In this section, we conducted experiments under the condition that model order equaled to 16 on a standard dataset of DNA sequences (Table 1), a DNA corpus consisting of four organisms used by Manzini et al. in [30] (Table 2), the bacteria DNA sequences (Table 3) from the National Center for Biotechnology Information (NCBI) directory, and the complete DNA sequences of ten species with various sizes (See Table 4). We represented time in seconds. In these experiments, the proposed compression algorithm without reference, i.e., COMPACT-NOREF, demonstrated performance advantage compared to existing models.

**Table 4:** Compression results on ten complete genomes.

Organism	Size	FCM-S (2011)	FCM-M (2011)		XM200 (2007)		COMPACT -seq		COMPACT -NOREF	
	MB	bpb	bpb	Time	bpb	Time	bpb	Time	bpb	Time
*H.sapiens*	2832.12	1.773	1.695	167 m	1.618*	958 m	1.749	742 m	1.673	2329 m
*A.thaliana*	119.48	1.911	1.821	165	1.659*	2820*	1.775	1454	1.776	3525
*A.nidulans*	29.54	1.987	1.978	38	1.968	717	1.952	424	**1.948**	871
*C.albicans*	14.32	1.882	1.864	17	1.861	291	1.836	198	**1.827**	500
*S.pombe*	12.59	1.926	1.887	15	1.865	358	1.861	173	**1.859**	440
*S.cerevisiae*	12.16	1.940	1.906	15	1.892	310	1.841	160	**1.838**	425
*E.coli*	4.64	1.937	1.901	5	1.914	48	1.903	54	**1.888**	152
*S.aureus*	2.80	1.888	1.858	4	1.852	31	1.847	33	**1.838**	95
*T.kodakarensis*	2.09	1.935	1.922	3	1.946	14	1.926	23	**1.902**	71
*M.jannaschii*	1.66	1.824	1.804	3	1.814	17	1.795	20	**1.791**	58
*M.genitalium*	0.58	1.841	1.812	2	1.816	3	1.806	6	**1.790**	19

Note: The minimum match length 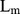 in the first pass is set to 25 in this experiment. The unit of time is second except for H.sapiens whose unit is minute. The ‘’XM200’’ column shows the results obtained with the XM algorithm using at most 200 experts. ‘*’ indicates that a model consumes much more memory (around or larger than 2.0 GB) than other methods.

### Performance of compression with reference

**Figure 8:**
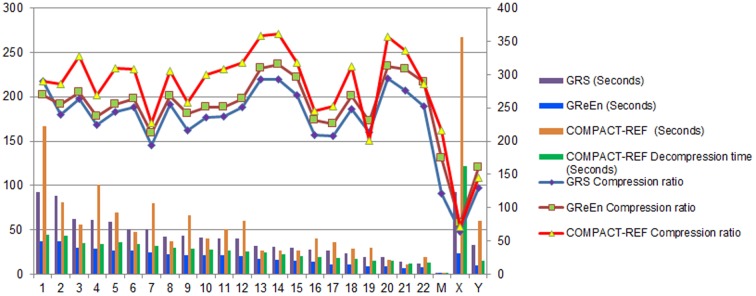
*Homo sapiens* genome: compression of KOREF_20090224 using KOREF_20090131 as reference. The original sequence alphabets have been preserved. The size of the alphabet in the target sequence is 21 for all chromosomes, except for chrM chromosome whose size is 11. The left y-axis refers to the compression ratio while the right y-axis indicates the compression or decompression time in seconds.

We tested the performance of COMPACT-REF (i.e. rLZ+COMPACT) with model order 16 and minimum match length 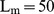 in three cases. As a result, the KOREF_20090224 genome sequence data using KOREF_20090131 as reference, for which the raw file is 2937.7 MB (KOREF_20090224), were compressed into a 15.8 MB file, achieving a 189-fold compression rate (Figure 8). Table 5 displays the 177-fold compression result for another experiment when the genome of a Han Chinese individual (YH) was compressed using KOREF_20090224 as reference. Table 6 displays the compression results of COMPACT-REF and GReEn [30] for three different human genome assemblies (YH, KOREF_20090224 and KOREF_20090131) with transformed alphabets using hg18 (NCBI36) as their common reference, and the results of them for the same datasets with original alphabets are displayed in Table S3 in File S1.

**Table 5:** *Homo sapiens* genome: compression of YH using KOREF_20090224 as reference.

Chr	Size	GRS(2011)		GReEn(2012)		COMPACT-REF			
	MB	MB	Time	MB	Time	MB	Time(c)	Time(d)	Offset size
1	235.80	-	-	2.24	34	1.40	1,115	58	[–12, 11560]
2	231.70	-	-	2.31	36	1.34	966	58	[–12, 11560]
3	190.26	16.60	651	1.65	29	1.06	465	45	[–12, 11560]
4	182.41	-	-	1.79	26	1.14	734	46	[–12, 11560]
5	172.48	-	-	1.71	25	0.95	783	43	[–12, 11560]
6	162.98	24.62	1,569	1.52	24	1.22	1,184	41	[–12,11560]
7	151.46	-	-	1.74	22	1.05	846	38	[–12, 11560]
8	139.50	-	-	1.30	21	0.94	100	35	[–12, 11560]
9	133.78	-	-	1.41	20	0.76	697	33	[–12, 11560]
10	129.10	-	-	1.29	19	0.71	630	32	[–12, 11560]
11	128.22	-	-	1.22	19	0.75	662	32	[–12, 11560]
12	126.22	15.39	3,016	1.12	19	0.82	716	31	[–12, 11560]
13	108.86	10.71	1,948	0.83	16	0.53	275	24	[–12, 11560]
14	101.44	-	-	0.79	15	0.55	454	25	[–12, 11560]
15	95.69	-	-	0.85	14	0.51	400	26	[–12, 11560]
16	84.71	-	-	0.97	12	0.50	164	22	[–12, 11560]
17	75.13	-	-	0.82	11	0.43	250	23	[–12, 11560]
18	72.59	12.58	2,245	0.68	11	0.43	210	17	[–12, 11560]
19	60.86	-	-	0.56	9	0.39	143	15	[–12, 11560]
20	59.54	8.02	510	0.47	8	0.36	231	15	[–12, 11560]
21	44.77	0.69	664	0.36	6	0.26	149	11	[–12, 11560]
22	47.39	-	-	0.42	9	0.25	144	13	[–12, 11560]
X	147.74	-	-	3.11	18	0.59	550	34	[–12, 11560]
Y	55.10	-	-	0.82	7	0.25	180	18	[–12, 11560]
M	16,571	321	1	127	1	274	1	1	[–12, 650]
Total	2937.73	-	-	29.96	428	16.57	12,049	736	-

Note: Since all bases in the original sequence alphabets are uppercase, we consider both exact repetitions and repetitions between the uppercase base and the respective lowercase base (e.g., ‘A’ and ‘a’). The missing values ‘-‘ of GRS method are due to the inability of GRS to compress sequences. The ‘Size’ unit of ChrM is Byte instead of MB. The unit of ‘Time’ is second. ‘Time(c)’ refers to the compression time and ‘Time(d)’ refers to the decompression time. In general, GReEn’s decompression time is identical to its compression’s.

**Table 6:** *Homo sapiens* genome: compression with COMPACT-REF and GReEn of the YH, KOREF_20090224 and KOREF_20090131 versions using hg18 as a reference.

chr	YH						KOREF24						KOREF31	
	*Size(MB)*		*Time(c)*		*Time(d)*		*Size(MB)*		*Time(c)*		*Time(d)*		*Size(MB)*	
	GR	CP	GR	CP	GR	CP	GR	CP	GR	CP	GR	CP	GR	CP
1	0.67	1.09	24	258	24	67	0.80	1.31	26	1,314	25	99	0.81	1.31
2	0.74	1.19	23	480	24	40	0.89	1.30	25	884	25	66	0.90	1.30
3	0.64	1.02	17	346	17	59	0.74	1.08	18	550	18	48	0.75	1.08
4	0.66	1.10	16	293	17	42	0.83	1.24	16	541	18	74	0.84	1.24
5	0.55	0.85	15	232	15	38	0.68	0.96	15	600	16	48	0.69	0.96
6	0.58	0.96	14	174	14	45	0.68	1.08	14	1,219	15	91	0.69	1.09
7	0.51	0.81	13	91	13	24	0.64	0.96	13	840	14	70	0.64	0.96
8	0.47	0.78	12	114	12	35	0.57	0.88	12	662	13	50	0.57	0.88
9	0.36	0.62	12	112	12	29	0.44	0.75	12	579	13	43	0.44	0.75
10	0.43	0.70	13	113	12	29	0.53	0.80	13	612	13	46	0.54	0.80
11	0.45	0.71	11	94	12	25	0.52	0.83	11	575	12	43	0.52	0.83
12	0.43	0.66	11	181	11	21	0.54	0.75	11	380	12	47	0.54	0.75
13	0.32	0.57	11	177	11	18	0.35	0.57	11	138	11	10	0.35	0.57
14	0.27	0.45	9	46	9	12	0.31	0.50	9	226	10	17	0.31	0.50
15	0.24	0.42	8	50	9	13	0.27	0.44	8	171	9	13	0.27	0.44
16	0.26	0.47	7	68	8	17	0.30	0.52	7	129	8	10	0.30	0.51
17	0.24	0.38	7	65	7	17	0.28	0.45	6	166	7	12	0.29	0.44
18	0.25	0.40	7	61	7	16	0.31	0.44	6	214	7	16	0.31	0.44
19	0.17	0.30	6	49	6	9	0.21	0.36	6	108	6	8	0.21	0.36
20	0.19	0.32	5	123	5	18	0.23	0.36	5	200	5	24	0.23	0.35
21	0.12	0.21	4	55	4	17	0.14	0.27	4	225	4	18	0.14	0.27
22	0.11	0.20	4	45	4	9	0.14	0.25	4	137	5	10	0.14	0.24
X	0.19	0.28	13	40	13	10	0.47	0.56	13	423	14	32	0.55	0.61
Y	0.02	0.03	4	23	4	6	0.06	0.18	4	222	5	17	0.07	0.19
T	8.84	14.53	266	3,290	270	616	10.91	16.84	269	11,115	285	912	11.08	16.88

Note: ‘T’ refers to ‘’Total’’. The unit of ‘Time’ is second. ‘Time(c)’ refers to the compression time and ‘Time(d)’ refers to the decompression time. ‘GR’ and ‘CP’ indicate the compared method ‘GReEn’ and the proposed ‘COMPACT-REF’ respectively. The compression/decompression time of KOREF31 which are omitted here approximately equal to that of KOREF24. For ease of comparison, we transformed all characters to lowercase and mapped all unknown nucleotides to ‘n’ before compression. After this transformation, all sequences were composed only of characters from the alphabet {a,c,g,t,n}.

## Discussion

### Discussion for compression without reference

Table 1 compares the compression results in bits per base (bpb). Along with our proposed algorithm, we presented here the existing algorithms, i.e. DNA3 [30], XM500 [13] and FCM [51]. Our COMPACT-NOREF with the proposed LZ method applied in the first pass outperformed the one with dnaX (a fast algorithm using fingerprints introduced by Manzini et al.[30]) for short sequences. But such advantage was not obvious as the sequence sizes increased. The reason is that encoding the finite repeats with a leading indicator rather than copious repeat locations saves space, and Gamma coding is more efficient than dnaX's continuation bit encoding for short sequences. Hence, we implemented the first pass of remaining experiments for reference-free scenarios with dnaX.

As for “difficult” sequences like HUMD (i.e., HUMDYSTROP), HUMH (i.e., HUMHBB) in which we did not gain performance advantage, we realized that both of them were human genomes, which often contained approximate repeats rather than exact duplicated strings. Therefore, the COMPACT-NONREF and other similar algorithms such as [23], [30], which only took the exact repeats into consideration, did not achieve the best performance for human genome compression. We have conducted a testing experiment in Figure S2 in File S1 to support this hypothesis. In Table 2, it is further witnessed that COMPACT-NOREF is a little inferior to XM for the “difficult” sequences of four organisms (i.e., yeast, mouse, arabidopsis, and human). However, we have realized that XM adopts more sophisticated modeling approach (e.g., the combination of various ‘’experts’’) and too many expert models to attain better representatoin for DNA sequences at a rapidly increasing cost of memory and time, while ours only picks out certain suitable ones relying on short-term knowledge from the past. In our experiments, XM took around or larger than 2.0 GB memory to compress the sequences over 100 MB in the forth group, which consumed much more resource than other methods (The proposed method took much less memory, please refer to Table S2 in File S1). We carried out additional experiments on the bacteria DNA sequences from the National Center for Biotechnology Information (NCBI) directory (Table 3). We compared a variation of the proposed algorithm: semi-COMPACT (i.e., COMPACT-NOREF without the first pass), COMPACT-NOREF, together with an XM encoder, and a state-of-the-art algorithm, FCM-Mx [28], in terms of compression and required time. Table 3 presents the individual compression results on these sequences with 10,000,000 or more bases. The table also includes the average compression result of each algorithm in the last row.

For bacteria, the proposed method demonstrated the best performance among all algorithms. The average compression rates of five sequences reported for XM500 and FCM-Mx were 1.787 bpb and 1.7543 bpb, while our method COMPACT-NOREF's average performance on the same set was 1.7204 bpb. The time cost for the proposed methods was comparable to that of XM. Results for eleven complete genomes are shown in Table 4. The FCM-S and FCM-M [52] columns contained results provided by the finite-context models and by the multiple competing finite-context models. FCM-S processed DNA sequences using the single finite-context model approach, in which the best context depth was used, whereas FCM-M obtained the results with the multiple competing models. The results presented in the Table show a similar pattern as Table 3. What’s more, all tables from 1 to 4 include the compression results of ‘COMPACT-seq’ that uses only traditional sequential models (including models based on Markov chains) followed by logistic regression model. Especially for Table 4, ‘COMPACT-seq’ outperforms all other algorithms on ten complete genomes, and ‘COMPACT-NOREF’ exceeds ‘COMPACT-seq’ on almost all sequences. It can be inferred that both the proposed non-sequential models and the logistic regression mixture model are extraordinary.

### Discussion for compression with reference

We compared the performance of the proposed method COMPACT-REF (i.e., rLZ+COMPACT) to that of GRS [15] and GReEn [17], two most recently proposed approach for compressing genome resequencing data that handled sequences over arbitrary alphabets. Figure 8 displays the compression performance for human genome KOREF_20090224 using KOREF_20090131 as reference. COMPACT-REF gave better results in terms of compression ratio but it is slower than GReEn and GRS. In fact, the speed disadvantage deserved a special note. Different from GReEn, the compression time of COMPACT-REF does not vary linearly with the size of the sequence but rather depending on the degree of similarity between the reference sequence and the target sequence. This was also the reason why some longer sequences took shorter time to compress, like chr8 and chr9. On the other hand, Figure 8 also demonstrates that COMPACT-REF can achieve comparable decompression consumption time with GReEn since both the decompression procedure and the decompression runtime of GReEn are identical to its compression’s, while COMPACT-REF saves time in the first pass for decompression because it only locates the match position instead of searching repeats. Hence, we believe that COMPACT-REF is advantageous in applications for which disk space and decompression time are the limiting factors but the compression time is more tolerable, such as sequence archive and sequence acquisition. In order to have a more specific and clearer comparison, we also present the results in tabular form in File S1 (Please refer to Table S1).

In order to provide a more comprehensive comparison between GRS, GReEn and the proposed compression approaches, we investigated another human genome assembly, YH, which referred to the genome of a Han Chinese individual. Table 5 displays the compression results of YH using KOREF_20090224 as reference. GRS performed poorly in both compression rate and speed. Our COMPACT-REF approach achieved good results with the appropriate window size, which can be selected by choosing a large window size and gradually shrinking it down. Note that the window range only slightly affects the compression performance. The default left window size and right window size are adaptively obtained through calculating the difference percentage, which equals to the sum of the difference values of each base’s (i.e., ‘A’, ‘C’, ‘T’, ‘G’, ‘N’, ‘a’, ‘c’, ‘t’, ‘g’ and ‘n’) number in the source sequence and reference sequence dividing by the base length of source sequence. If the percentage is smaller than 0.65%, the default window size is set to [–12, 650]; If the percentage is larger than 0.65% but smaller than 5%, the default window size is set to [–12, 812]; Otherwise the window size is set to [–12, 11560]. For the compression of KOREF_20090224 using KOREF_20090131 as reference, the default window range is [–12,650] except for [–12, 812] for chr1, 4, X and chrY. In the compression of YH using KOREF_20090224 as reference, the default window range is [–12, 11560] for most chromosomes.

Table 6 summarizes compression results of COMPACT-REF and GReEn for three different human genome assemblies (YH, KOREF_20090224 and KOREF_20090131) using the same choice of reference hg18 (NCBI36). KOREF_20090131, KOREF_20090224 and YH database are three genome databases generated by two different organizations. YH is the first diploid genome sequence of a Han Chinese, a representative of Asian population, completed by Beijing Genomics Institute at Shenzhen (BGI-Shenzhen). KOREF_20090131 and KOREF_20090224 are two versions of the first individual Korean genome released in December 2008 as the result of Korean reference genome construction project. Consequently, the alphabet set of these two datasets are different. Both KOREF_20090131 and KOREF_20090224 consist of 21 symbols, such as ‘A’, ‘C’, ‘T’, ‘G’, ‘N’, ‘M’ and etc., with the additional bases besides {‘A’, ‘C’, ‘T’, ‘G’} indicating different sequencing quality or uncertainty. But all bases in YH are confined to {‘A’, ‘C’, ‘T’, ‘G’, ‘N’} by using only ‘N’ to represent uncertain bases. Hence, in order to keep the alphabet size identical, we transformed all characters to lowercase and mapped unknown nucleotides to 'n' for the sake of comparison. Table S3 in File S1 displays the compression results of COMPACT-REF and GReEn for the same datasets with original alphabets. The size of each sequence in Table 6 reduced significantly by the proposed method although GReEn seems to show a superior performance. The reason why GReEn generates better compression results than COMPACT-REF in this situation may be that GReEn relies on the probability distribution of characters in the target sequence (assuming that the characters of the target sequence are an exact copy of (parts of) the reference sequence). When we do not eliminate the effect of character case (i.e., uppercase or lowercase) in Table S3, GReEn demonstrates an obvious disadvantage in terms of compression performance. These experiments demonstrated the applicability of our framework in compressing genomic data sets and encouraging further investigation.

## Supporting Information

File S1 **Supporting figures and tables. Figure S1.** The diagram of logistic regression model synthesizing different models to obtain a single probability. **Figure S2**. The relationship between the compression rate and the quantity of noise over the sequence HEHCMVCG. **Figure S3**. The schematic diagram of the selected contexts for eleven non-sequential sparse models. Red block refers to the picked bit while the others refer to the excluded one. **Table S1**. Homo sapiens genome: compression of KOREF_20090224 using KOREF_20090131 as reference. **Table S2**. The evalution of memory usage in our experiments. **Table S3**. Homo sapiens genome: compression with COMPACT-REF and GReEn of the YH, KOREF_20090224 and KOREF_20090131 versions with original alphabets using hg18 as a reference.
(DOCX)
