# Supplementary material for: DNA-COMPACT: DNA COMpression Based on a Pattern-Aware Contextual Modeling Technique
Source: PLoS One. 2013 Nov 25;8(11):e80377. doi: 10.1371/journal.pone.0080377 (PMC3840021; doi:10.1371/journal.pone.0080377)
Supplement: File S1 — Supporting figures and tables. Figure S1. The diagram of logistic regression model synthesizing different models to obtain a single probability. Figure S2. The relationship between the compression rate and the quantity of noise over the sequence HEHCMVCG. Figure S3. The schematic diagram of the selected contexts for eleven non-sequential sparse models. Red block refers to the picked bit while the others refer to the excluded one. Table S1. Homo sapiens genome: compression of KOREF_20090224 using KOREF_20090131 as reference. Table S2. The evalution of memory usage in our experiments. Table S3. Homo sapiens genome: compression with COMPACT-REF and GReEn of the YH, KOREF_20090224 and KOREF_20090131 versions with original alphabets using hg18 as a reference. (DOCX) [file pone.0080377.s001.docx]

**Appendix 1**

We added one diagram here to show how logistic regression model works. The entire scheme aims at training the weights on individual model (weak ones, $t_{1},t_{2}\cdots,t_{M_{c}}$) to obtain a strong one, i.e., $P(y_{j}|t_{1}^{M_{c}})$. Like the famous Adaptive Boost (AdaBoost[[1]](#footnote-1)) algorithm, the logistic regression is capable of combining weak classifiers to obtain a strong one.


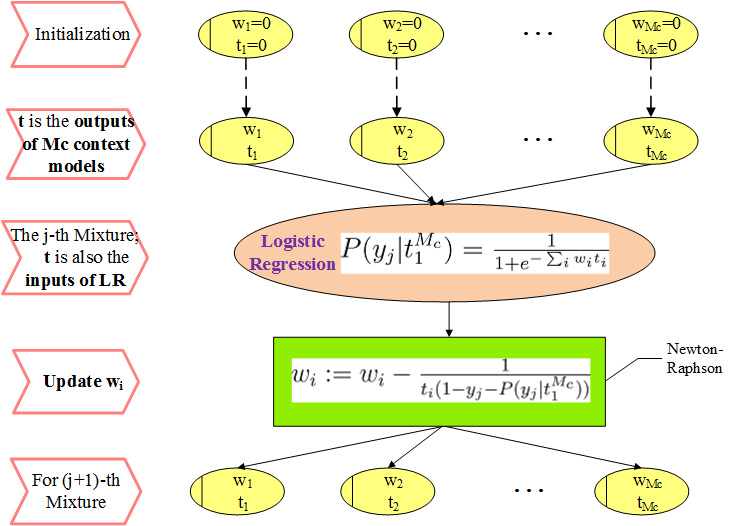


Figure S1. The diagram of logistic regression model synthesizing different models to obtain a single probability.

The computational overhead of fitting the logistic regression model in general applications mainly roots in the complicated computation and iteration of Newton’s (i.e., Newton-Raphson) method. For instance, each process begins with a tentative solution, revises it slightly to see if it can be improved, and repeats this revision until improvement is minute, at which point the process is said to have converged. However, the logistic regression model in our case does not need to go through these procedures for each bit. Combined with Figure S1, we provide the derivation process of weights based on Newton’s method.

In Equation (5) of main text, we obtained the synthesized probability of the predicted bit $y_{j}$ equaling to 1:

$$P(y_{j}|t_{1}^{M_{c}})=\frac{1}{1+e^{-\sum_{i} w_{i}t_{i}}}$$

After bit $y_{j}$ (0 or 1) is received, we obtain the prediction error $E=y_{j}-P(y|t)$. The goal in compression is to minimize coding cost, i.e.,

$$\begin{matrix} \min_{w_{i}} & C \end{matrix}=\begin{matrix} \min& -\ln(1-|E|)=\left\{\begin{matrix} -\ln(\frac{1}{1+e^{-\sum_{i} w_{i}t_{i}}})\begin{matrix} & \begin{matrix} for & y_{j}=1 \end{matrix} \end{matrix} \\ -\ln(\frac{e^{-\sum_{i} w_{i}t_{i}}}{1+e^{-\sum_{i} w_{i}t_{i}}})\begin{matrix} & \begin{matrix} for & y_{j}=0 \end{matrix} \end{matrix} \end{matrix}\right. \end{matrix}$$

Based on Newton-Raphson’s method, we take the partial derivative and second order partial derivative of cost with respect to $\text{w}_{\text{i}}$ respectively, and obtain the update rule for $\text{w}_{\text{i}}$:

$$\text{w}_{\text{i}}:=\text{w}_{\text{i}}-\frac{\frac{\partial C}{\partial\text{w}_{\text{i}}}}{\frac{\partial^{2}C}{\partial\text{w}_{_{\text{i}}}^{2}}}=\text{w}_{\text{i}}-\frac{1}{t_{i}(1-y_{j}-P(y_{j}|t_{1}^{M_{c}}))}$$

For each context model, we only apply this simple equation once to update its weight $w_{i}$ and the updated weight will be used for the prediction of next bit. Gradually, the weights are adjusted to fit the input sequence. Hence, the complicated computation and iteration problem of Newton’s method in other cases are eliminated in our method.

**Appendix 2**

To support the hypothesis that it was on account of the excessive non-exact repeatives in human genomes that the proposed algorithm obtained worse results than XM500, we made a test using non-human sequences polluted with noises.

We generated polluted data by randomly changing some bases into their complementary bases (e.g., ‘A’ with ‘T’ and ‘C’ with ‘G’ are complementary to each other). We took the non-human sequence HEHCMVCG (length = 229354) as an example. The relationship between the compression rates of ‘XM500’ and our proposed ‘COM-NONREF’ and the quantity of noise that ranges from 0 to 3000 is depicted in Figure S1. Although COM-NONREF demonstrates great advantage over XM500 for the original sequence (i.e., the quantity of noise equals to 0), it is obvious that the compression rate of our proposed algorithm increases more than that of XM500 as the quantity of noise increases. Especially, when there are 2500 random perturbations in the sequence, the gap of the compression rates between XM500 and the proposed method becomes relatively small. And when the number of random perturbations reaches 3000, the proposed method obtains worse performance than XM500. Consequently, it is understandable that XM500 can provide better results than our proposed algorithm for human genomes due to their excessive non-exact copies.

Figure S2. The relationship between the compression rate and the quantity of noise over the sequence HEHCMVCG.

**Appendix 3**

In order to have a more specific and clearer comparison, we presented the results in Figure 8 in tabular form.

Table S1: Homo sapiens genome: compression of KOREF_20090224 using KOREF_20090131 as reference.

| Chr | Size | GRS(2011) | | GReEn(2012) | | COMPACT-REF | | |
| --- | --- | --- | --- | --- | --- | --- | --- | --- |
|  | MB | Ratio | Time | Ratio | Time | Ratio | Time(c) | Time(d) |
| 1 | 235.80 | 217.53 | 124 | 201.71 | 50 | 217.05 | 223 | 59 |
| 2 | 231.70 | 179.42 | 118 | 190.98 | 49 | 214.17 | 108 | 58 |
| 3 | 190.26 | 197.31 | 83 | 205.35 | 40 | 245.91 | 75 | 47 |
| 4 | 182.41 | 167.90 | 81 | 178.03 | 38 | 202.25 | 135 | 45 |
| 5 | 172.48 | 183.04 | 78 | 190.90 | 36 | 232.07 | 92 | 49 |
| 6 | 162.98 | 188.61 | 68 | 197.47 | 35 | 230.82 | 63 | 45 |
| 7 | 151.46 | 144.82 | 68 | 159.06 | 32 | 170.83 | 106 | 43 |
| 8 | 139.50 | 191.38 | 57 | 200.55 | 30 | 228.65 | 50 | 39 |
| 9 | 133.78 | 162.31 | 58 | 181.30 | 29 | 193.25 | 89 | 38 |
| 10 | 129.10 | 176.19 | 55 | 188.73 | 28 | 225.13 | 54 | 37 |
| 11 | 128.22 | 177.92 | 53 | 187.70 | 28 | 231.46 | 67 | 36 |
| 12 | 126.22 | 188.52 | 53 | 197.99 | 27 | 238.71 | 80 | 34 |
| 13 | 108.86 | 219.25 | 42 | 232.52 | 23 | 268.50 | 36 | 32 |
| 14 | 101.44 | 219.41 | 41 | 235.84 | 21 | 270.75 | 36 | 30 |
| 15 | 95.69 | 202.21 | 39 | 221.35 | 20 | 238.00 | 36 | 27 |
| 16 | 84.71 | 156.39 | 37 | 174.08 | 18 | 184.46 | 53 | 26 |
| 17 | 75.13 | 155.69 | 35 | 169.65 | 15 | 188.78 | 48 | 24 |
| 18 | 72.59 | 186.32 | 31 | 201.14 | 14 | 234.34 | 38 | 23 |
| 19 | 60.86 | 159.61 | 26 | 172.75 | 12 | 150.10 | 39 | 20 |
| 20 | 59.54 | 220.91 | 26 | 234.23 | 12 | 267.83 | 21 | 20 |
| 21 | 44.77 | 207.21 | 19 | 231.21 | 9 | 252.24 | 15 | 16 |
| 22 | 47.39 | 189.34 | 16 | 216.00 | 10 | 214.14 | 26 | 17 |
| M | 0.0158 | 90.55 | 1 | 130.48 | 1 | 162.46 | 0.32 | 0.1 |
| X | 147.74 | 47.93 | 124 | 57.12 | 31 | 53.37 | 356 | 163 |
| Y | 55.10 | 97.46 | 44 | 120.03 | 13 | 108.86 | 80 | 20 |

Note: The unit of ‘Time’ is second. ‘Time(c)’ refers to the compression time and ‘Time(d)’refers to the decompression time.

**Appendix 4**

The memory of our method is mainly consumed by the searching of appropriate repeats and the representation of the context models. For example, the memory will increase exponentially as the model order grows if we store the information of each state of the contexts. There is a tradeoff between the runtime and memory consumption. We chose to sacrifice a little time to save much memory so that the memory cost in our experiments is remarkable small. We have included some representative experimental results of memory usage in Table S2.

Table S2 The evalution of memory usage in our experiments.

| Sequence | Size | Scheme | Memory used for Compression | Memory used for Decompression |
| --- | --- | --- | --- | --- |
| genitalium | 580,076 | COMPACT-NONREF | 121MB | 121MB |
| chr22 of YH | 49,691,432 | COMPACT-REF | 125MB | 125MB |
| h-22 | 33,821,688 | COMPACT-NONREF | 175MB | 175MB |
| chr1 of KOREF_20090224 | 247,249,719 | COMPACT-REF | 237MB | 151MB |

**Appendix 5**

We did not take into consideration all possible non-sequential context models. Indeed, there are a total of 2^32^-32 (i.e., 4294967264) different non-sequential context models, which perform on the last four bytes ahead of the base including the bit to be compressed. In principle, we should customize the optimal combination of models under the minimum description length criterion. That is, in order to determine the optimal combination of possible proper context models, context models should be evaluated by comparing the performance gain when they are included or excluded in the prediction. However, it is infeasible to scan all non-sequential context models due to their computational complexity. To cut down the overall computational cost and make it computationally feasible, we have to pick a sample from context models. We conducted a random symmetrical selection models from complementary non-sequential models to ensure the number of selected bits approximately identical to the number of excluded ones (see the following figure). In the end, we use the sparsest models that demonstrated low complexity and good compression performance.


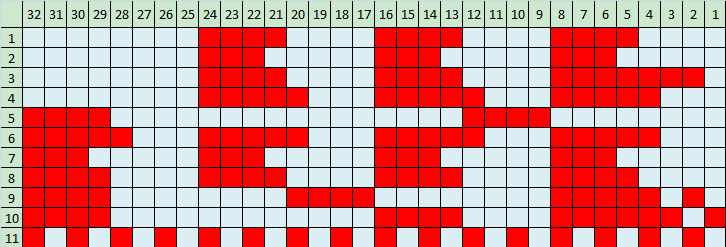


Figure S3. The schematic diagram of the selected contexts for eleven non-sequential sparse models. Red block refers to the picked bit while the others refer to the excluded one.

**Appendix 6**

In order to provide a comprehensive comparison between GReEn and COMPACT-REF on the compression of YH, KOREF_20090224 and KOREF_20090131 using hg18 as a reference, we presented the compression results with three datasets’ original alphabets in the following table while keeping Table 6 intact in the main text. Because GReEn relies on the probability distribution of characters in the target sequence (assuming that the characters of the target sequence are an exact copy of (parts of) the reference sequence), it demonstrates an inferior performance when we do not eliminate the effect of character case (i.e., uppercase or lowercase).

Table S3 *Homo sapiens* genome: compression with COMPACT-REF and GReEn of the YH, KOREF_20090224 and KOREF_20090131 versions with original alphabets using hg18 as a reference.

| chr | YH | | | | | | KOREF24 | | | | | | KOREF31 | |
| --- | --- | --- | --- | --- | --- | --- | --- | --- | --- | --- | --- | --- | --- | --- |
|  | *Size(MB)* | | *Time(c)* | | *Time(d)* | | *Size(MB)* | | *Time(c)* | | *Time(d)* | | *Size(MB)* | |
|  | GR | CP | GR | CP | GR | CP | GR | CP | GR | CP | GR | CP | GR | CP |
| 1 | 17.7 | 1.1 | 29 | 258 | 32 | 67 | 18.5 | 2.6 | 42 | 809 | 44 | 125 | 18.6 | 2.9 |
| 2 | 18.3 | 1.2 | 29 | 480 | 32 | 40 | 19.3 | 2.6 | 42 | 749 | 44 | 110 | 19.4 | 2.9 |
| 3 | 14.9 | 1.0 | 23 | 346 | 25 | 59 | 15.8 | 2.1 | 35 | 590 | 36 | 80 | 15.9 | 2.4 |
| 4 | 13.9 | 1.1 | 22 | 293 | 23 | 42 | 14.9 | 2.3 | 33 | 785 | 34 | 115 | 15.0 | 2.6 |
| 5 | 13.8 | 0.9 | 22 | 232 | 22 | 38 | 14.7 | 1.9 | 31 | 550 | 33 | 82 | 14.7 | 2.1 |
| 6 | 12.7 | 1.0 | 20 | 174 | 21 | 45 | 13.4 | 2.0 | 29 | 860 | 30 | 95 | 13.5 | 2.3 |
| 7 | 11.9 | 0.8 | 19 | 91 | 20 | 24 | 12.5 | 1.9 | 27 | 600 | 28 | 90 | 12.6 | 2.2 |
| 8 | 11.1 | 0.8 | 17 | 114 | 17 | 35 | 11.7 | 1.7 | 25 | 530 | 26 | 85 | 11.8 | 1.9 |
| 9 | 9.6 | 0.6 | 16 | 112 | 17 | 29 | 9.9 | 1.5 | 23 | 435 | 25 | 68 | 9.9 | 1.7 |
| 10 | 10.3 | 0.7 | 16 | 113 | 16 | 29 | 10.8 | 1.6 | 24 | 445 | 25 | 70 | 10.8 | 1.7 |
| 11 | 9.9 | 0.7 | 16 | 94 | 16 | 25 | 10.5 | 1.6 | 23 | 495 | 24 | 70 | 10.6 | 1.8 |
| 12 | 10.2 | 0.7 | 15 | 181 | 16 | 21 | 10.7 | 1.5 | 23 | 402 | 23 | 62 | 10.8 | 1.6 |
| 13 | 7.0 | 0.6 | 13 | 177 | 14 | 18 | 7.5 | 1.1 | 19 | 300 | 20 | 50 | 7.5 | 1.3 |
| 14 | 6.7 | 0.4 | 12 | 46 | 13 | 12 | 7.0 | 1.0 | 18 | 269 | 18 | 40 | 7.1 | 1.1 |
| 15 | 6.3 | 0.4 | 12 | 50 | 12 | 13 | 6.6 | 0.9 | 17 | 231 | 18 | 32 | 6.7 | 1.0 |
| 16 | 6.6 | 0.5 | 10 | 68 | 11 | 17 | 6.9 | 1.1 | 15 | 325 | 16 | 51 | 6.9 | 1.2 |
| 17 | 6.5 | 0.4 | 10 | 65 | 10 | 17 | 6.8 | 0.9 | 14 | 271 | 13 | 42 | 6.8 | 1.0 |
| 18 | 5.6 | 0.4 | 9 | 61 | 9 | 16 | 5.9 | 0.9 | 13 | 235 | 13 | 38 | 6.0 | 0.8 |
| 19 | 4.5 | 0.3 | 7 | 49 | 8 | 9 | 4.7 | 0.8 | 11 | 295 | 12 | 50 | 4.7 | 0.9 |
| 20 | 4.9 | 0.3 | 7 | 123 | 7 | 18 | 5.2 | 0.7 | 11 | 210 | 11 | 32 | 5.2 | 0.7 |
| 21 | 2.6 | 0.2 | 5 | 55 | 6 | 17 | 2.7 | 0.5 | 8 | 160 | 7 | 25 | 2.7 | 0.5 |
| 22 | 2.8 | 0.2 | 6 | 45 | 6 | 9 | 3.0 | 0.5 | 8 | 158 | 9 | 24 | 3.0 | 0.5 |
| X | 12.7 | 0.3 | 15 | 40 | 16 | 10 | 13.2 | 2.5 | 27 | 501 | 27 | 80 | 13.3 | 3.9 |
| Y | 2.5 | 0.0 | 5 | 23 | 6 | 6 | 2.4 | 0.5 | 9 | 321 | 10 | 51 | 2.3 | 0.7 |
| T | 223.0 | 14.5 | 355 | 3,290 | 375 | 616 | 234.6 | 34.6 | 527 | 10,205 | 546 | 1567 | 235.8 | 39.8 |

Note: ‘T’ refers to ‘’Total’’. The unit of ‘Time’ is second. ‘Time(c)’ refers to the compression time and ‘Time(d)’ refers to the decompression time. ‘GR’ and ‘CP’ indicate the compared method ‘GReEn’ and the proposed ‘COMPACT-REF’ respectively. The compression/decompression time of KOREF31 which are omitted here approximately equal to that of KOREF24. The original sequence alphabets have been preserved.

1. <http://en.wikipedia.org/wiki/AdaBoost> [↑](#footnote-ref-1)
